# How Does Family Intergenerational Relationships Affect the Life Satisfaction of Middle-Aged and Elderly Parents in Urban Only-Child Families in Chengdu, China

**DOI:** 10.3390/ijerph19148704

**Published:** 2022-07-17

**Authors:** Tonggang Zeng, Yongchun Yang, Shan Man

**Affiliations:** 1 College of Earth and Environmental Sciences, Lanzhou University, Lanzhou 730000, China; tonggangzeng@163.com (T.Z.); manshan0725@163.com (S.M.); 2 Key Laboratory of Western China’s Environmental Systems (Ministry of Education), Lanzhou University, Lanzhou 730000, China

**Keywords:** only-child, family intergenerational relationships, gender, life satisfaction, aging

## Abstract

Over the past 40 years, the implementation of the family planning policy in China has led to the creation of many only-child families. In the process of modernization and urbanization, it is critical to focus on the intergenerational relationships in only-child families and their associational mechanism on the life satisfaction of middle-aged and elderly parents, which has crucial implications for them staying active and healthy aging. Using the survey data from Chengdu, China, this study analyzed the characteristics of only-child parents’ life satisfaction and family intergenerational relationships, and explored the associational mechanism of family intergenerational relationships on only-child parents’ life satisfaction in urban families, as well as the possible moderating role of gender. The results indicate that there are gender differences in the life satisfaction of only-child parents in urban families, and men are more satisfied than women. Moreover, parents of sons and daughters differ in life satisfaction from the dual-gender perspective. Parents of daughters are likely to have higher life satisfaction, especially mothers. The only-child families have not moved toward nucleation in urban families, and intergenerational members maintain close contact and provide frequent mutual support to achieve individual and family development. There are significant gender differences in structural, associational, affectual, and functional solidarity among only-child. This study confirms that there are differences in the associational mechanism of family intergenerational relationships on life satisfaction in different dimensions. Affectual solidarity is the most influential factor of life satisfaction. In terms of normative and consensual solidarity, gender plays a moderating role. For men, normative and consensual solidarity is beneficial for improving life satisfaction, but it has an insignificant effect on women. The effects of structural solidarity, association solidarity, and functional solidarity are not significant.

## 1. Introduction

Owing to a rapid economic development and its accompanying social changes in contemporary China, the scale, structure, and function of the Chinese family, as well as family relations, have undergone considerable changes. In the process of social transformation, the superposition of state systems has boosted the demographic and family transition in China, which has unconventional properties [1]. Since 1973, the Chinese government had advocated the implementation of the family planning policy, with the main guideline of “late, rare, and few”. In 1980, the one-child policy began to be implemented in urban areas, which meant that China’s one-child policy was formally introduced to curb population growth [2]. The full implementation of the family planning policy led to a continuous decline in the total fertility rate of the population, and only-child families formed on a large scale, especially in Chinese cities. In reality, the “421” inverted pyramid family structure, the fragility and variability of the only-child family, and the enormous pressure on family development promoted the timely adjustment and optimization of China’s social policies. In 2013, the introduction of the “Conditional two-child” policy marked the loosening of the strict fertility policy. In 2016, the implementation of the “Universal two-child” policy announced the end of the only-child policy in China. Since the implementation of the one-child policy in the 1970s, the first generation of only-child parents are going to or have entered the elderly population cohort in China. For a long time, China has mainly relied on the family pension model, which has always played a fundamental role in China’s pension system [3]. However, multiple complex factors such as modernization, urbanization, and industrialization are constantly changing the traditional family intergenerational relationships. Therefore, in the process of a rapid social transformation, how have the intergenerational relationships of Chinese one-child families changed? The impact of changes in family intergenerational relationships on the survival and development of middle-aged and elderly parents has undoubtedly become a key issue that needs to be considered in the process of active and healthy aging.

In early family studies in Western society, the classic family modernization theory represented by Parsons and Goode [4] holds that in the process of modernization, traditional families tend to become nucleus and nonkinship, and undergo defunctionalization. The assumption of a one-way path and linear process of family transition ignores the complex interaction effects between the family and the external support system. In fact, the Western model, with homogeneity as the core, is difficult to use to explain the complexity of family transformations, and the diversity of family forms has become a consensus in the academic community [5,6]. Many studies [6,7,8], including case studies from European and American countries, have shown that extended kinship family networks still exist in industrial and postindustrial societies, which have shifted from the previous emphasis on modern individualism to refocus on the positive role of kinship networks in traditional families. In this context, Bengtson put forward the theoretical framework of intergenerational solidarity and its measurement model in the 1970s [9], which draws on the thinking of family integration and provides a better framework for explaining the complex and diverse experiences and behaviors in family intergenerational relations. Based on this theoretical framework, many scholars have analyzed countries or regions with different cultural and economic backgrounds, such as European and American countries [10,11], East Asian countries [12,13], and Taiwan [14], which has become the dominant paradigm in most studies on family intergenerational relations. China is a home-based society [15]. In the traditional society, the Confucian filial piety culture and the corresponding social and cultural norms play a positive role in family intergenerational relations. With the implementation of the reform and opening-up policy and the development of the market economy in the 1980s, most Chinese local studies have adopted the methodology of the modernization theory to analyze the changes and transformations of intergenerational family relations in China, constantly verifying and challenging Western theories on family intergenerational relations. Some scholars, such as He [16] and Wang et al. [17], argue that there is an imbalance in the intergenerational relationships in rural China. More specifically, urbanization has disintegrated the patriarchal family system, weakened the parental authority, and the constraining role of the original kinship network. Accordingly, the traditional “feedback model” has also begun to decline. There are also some scholars, such as Cong [18], Izuhara [19], and Yan [20], who have concluded that the family intergenerational relationship represents the fusion and confrontation of tradition and modernity, which is contrary to the assumed mode of modernization. It contains the dynamism of family development and undergoing diversification, whereas the extended family network is highly integrated into social relations. There are frequent intergenerational interactions in the family, and intergenerational cooperation and reciprocity still exist widely. In addition, Liu [21] and Wu [22] also reached a similar conclusion in their specialized studies on the only-child families in China.

Close family intergenerational relationships as an abstract and multidimensional concept are characterized by frequent contact, high emotional closeness, and high filial duty [23,24]. The living arrangement is the external manifestation of the family intergenerational relationships, and it has become the most direct observation dimension [24,25]. Observations from developed and less developed countries, including China, have confirmed a declining proportion of intergenerational cohabitation [26,27]. In contrast to Western countries, China’s traditional extended family model contains deep social norms about filial piety and intergenerational obligations. Under this logic, the decline in the number of multigenerational households and the migration of the labor force may be disrupting traditional living arrangements, which may continue to impact the traditional Chinese family care function and elderly well-being. Most studies believe that living arrangements have a significant effect on elderly people’s mental health [28], mortality risk [29], and life satisfaction [30]. Where traditional culture is more prevalent, older adults are more likely to live with adult or married sons in China [31]. While some studies have found that, compared with living with a son, living with a daughter has a more positive effect on the psychological well-being of older adults [32]. In addition, some studies have demonstrated that multigenerational living is more likely to cause intergenerational conflict [33], thus, leading to negative effects that may offset or even outweigh the positive effects of other aspects.

In the discussion of intergenerational support, there are also large differences in the analysis conclusions of different scholars regarding the type of intergenerational support, such as economic, emotional, and instrumental support, and the gender of the adult children. Most of these studies have focused on the elderly in multichild families, ignoring the differences in different stages of life development and family structures. Based on the analysis of the effect of spatial separation, some scholars believe that the intergenerational separation of parents and adult children weakens family support for the rural elderly from the aspects of daily care and spiritual comfort, which are restricted by space, but it strengthens the economic support, which is not restricted by space in China [25]. In addition, the elderly who maintained positive intergenerational relationships with their children would prompt the elderly to obtain adequate family social support [34]. The cultural context affects the way persons deal with the norm of reciprocity. In most Western countries, there is no obvious difference in the gender structure of children’s filial piety expectations of parents [35]. However, in traditional Chinese society, where women are in a dependent position in the family, adult daughters play an auxiliary role, rather than an alternative role in family support. A daughter’s participation in filial piety may be more out of close intergenerational affection, rather than the son’s obligatory and utilitarian motives [18,36]. Analyses by adult children’s gender suggest that gender differences between sons and daughters in intergenerational support have reduced, and daughters are even more beneficial to their parents [37,38], subject to the influence of traditional gender culture. Meanwhile, with only a few studies of only-child families in China, which believe that the gendered pattern of intergenerational support has not changed, sons were more likely to receive family support from elderly parents [39]. There were gender differences in the association between intergenerational support and life satisfaction for the elderly, for both exchange patterns and different types of support. Analyses by parents’ gender show that older mothers receive more returns, which reciprocates their support, while older mothers depend more on their children due to their disadvantages in economic status and health [40]. In order to adapt to the urban–rural mobility of adult children, Chinese rural parents may adjust their filial piety expectations, generally expecting their children to express their filial piety in some reasonable way, such as financial support, so as to improve their life satisfaction [15]. A lack of intergenerational support and interaction was a risk factor associated with depression [3,34], cognitive impairment [41], and reduced their adaptability to aging [32]. However, some studies have suggested that support from children weakens the self-utility evaluation of the elderly, leading to greater health risks [42]. Filial piety plays an important role in family relationships in Chinese culture. A study on filial piety identification in China found that both the number of daughters and having at least one daughter could significantly reduce the risk perception of filial piety of urban parents [43]. In addition, Chinese daughters are more likely to identify with intergenerational reciprocity motivations than German daughters in cross-cultural comparative research [44].

Different from the above studies, this study aimed to address the following points: (1) In the context of fewer children and the aging population, this study used the intergenerational solidarity theory to compare the possible differences in multidimensional relationships, which reflect the characteristics of intergenerational relations in the process of the modernization of only-child families in urban China. (2) During the social transition period, how do changes in the intergenerational relationships of only-child families in urban China affect the life satisfaction of middle-aged and elderly parents? Are there different effects of different gender subjects? The purpose of this study was to grasp the transformation of the intergenerational relationship of the urban only-child family in China during the process of modernization and analyze its associational mechanism on the life satisfaction of the middle-aged and elderly from the perspective of intergenerational solidarity, which would be helpful in responding to the existence of the traditional family pension model. The study is expected to provide a certain scientific basis for the Chinese society to actively respond to the aging of the population and build an age-friendly society.

## 2. Research Methods and Data Sources

### 2.1. Study Design

In order to answer the research questions, mixed methods research was used in this study [45]. The aim was that, considering the complexity and diversity of the research objects, this study tried to use both qualitative and quantitative research methods to maximize the realization of research objectives. The embedded design method was adopted in this study [46]. Its research path was mainly to embed the qualitative part in the design of traditional quantitative research, so as to enhance the validity and stability of the research conclusions. In this research methodology, open-ended interview data and quantitative questionnaire data were collected and processed in this study. Based on the quantitative research, the textual materials obtained from the qualitative research were used to interpret or supplement the results of the quantitative analysis and to cross-validate the findings, which helped to develop a relatively complete knowledge of the research questions. This study was primarily based on a quantitative research design to make decisions on the implementation of qualitative research, so the quantitative and qualitative components of the study could be regarded as interactively related [46].

This was a mixed study with two phases. In the first phase, qualitative data were collected using a semistructured interview method. Based on demographic characteristics, the research team recruited 15 interviewees for semistructured interviews in May 2021, each lasting 30–60 min. In addition to the basic information of the interviewees, the qualitative interviews focused on the intergenerational interaction characteristics, values, and behavioral motivations in some specific life events. On this basis, combined with the results of the semistructured interviews, the research team carried out multiple rounds of revisions and adjustments to the questionnaire, which was needed in the next phase. In the second phase, the research team conducted a questionnaire survey from May to July 2021. In addition to the 15 interviewees who completed the questionnaire in the first phase, new participants continued to be recruited into the questionnaire phase of this study. All participants were asked to complete a questionnaire either independently or with the help of a researcher (just explaining the questions, but not interfering with their answers).

All participants in both phases were recruited using random sampling and snowball sampling. This study strictly followed the principle of ethical confidentiality and provided detailed information to the research subjects about the purpose, significance, and methods of this study, as well as their rights as research subjects and measures to protect their privacy. All data collection was performed with the informed consent of the study subjects. Once again, the research group would like to thank all of these respondents for participating in this study.

### 2.2. Case Selection and Data Collection

The data were derived from “The Survey on the Living Conditions of Only-Child Families in the Period of Social Transition” conducted by the research group from May to July 2021, which selected Chengdu, Sichuan province, as the case site, including five central urban areas, namely, Jinjiang, Qingyang, Jinniu, Wuhou, and Chenghua districts. The respondents of this study were parents of only one child. Regarding the definition of an only-child, this study referred to the existing research [22], which defines it as a person born between 1975 and 1995 and having no siblings, while their parents are defined as only-child parents. Due to the constraints of the sampling frame of only-child families in Chengdu, this study used statistical data, namely, “Number of only-child aged 0–30 by region” in the “National 1% Sample Survey Data” (2005) and “Sichuan Province 1% population Sample Survey Data” (2015) to ensure that the sample data were representative and valid. A total of 450 questionnaires was distributed with the simple random sampling method, and 408 valid questionnaires were reviewed (including the 15 interviewees from the first phase) made up of 82 from the Jinjiang district, 78 from the Qingyang district, 85 from the Jinniu district, 76 from the Wuhou district, and 87 from the Chenghua district. Thus, the questionnaire’s efficiency was 90.67%. Demographic characteristics are shown in Table 1.

Chengdu was chosen as the case site for two reasons. One reason was the local peculiarity in the implementation of the fertility policy. In the late 1980s, there were large regional and urban–rural differences in the implementation of China’s fertility policy. The one-child policy was strictly implemented in urban areas, except rural areas in Sichuan, Jiangsu, and Chongqing, while in most other rural areas, if a couple’s first-born was a girl, they were permitted to have a second child [47,48]. On the whole, Sichuan province basically strictly implemented the only-child policy of “Han residents have one child per couple”, which provided a relatively ideal research case in this study. The second reason was that Chengdu, as a place where traditional culture and modern civilization are intertwined in China, is located in the western part of the Sichuan Basin and on the eastern edge of the Qinghai–Tibet Plateau. The superior geographical conditions have enabled its long history. Chengdu is an ancient city with a long history of more than 2300 years, and has been known as the “Land of Abundance” since ancient times. Chengdu is also a modern city. Chengdu is the provincial capital of Sichuan province, a subprovincial city and a megacity in China, and is one of the core cities of the “Sichuan-Chongqing economic circle”. In 2020, Chengdu achieved a gross regional product (GDP) of 1771.67 billion CNY, and the per capita disposable income of urban residents was 48,593 CNY. The city’s resident population is 20.9378 million, and the urbanization rate is 78.77%. The floating population is 8.4596 million. In the whole year, the total foreign trade import and export volume was 715.42 billion CNY, and there were 305 Fortune 500 enterprises in Chengdu.

### 2.3. Variable Description and Model Selection in Quantitative Research

This study took the urban family intergenerational relationship as the starting point of research. Referring to the intergenerational solidarity theory [9,13], this study divided the family intergenerational relationship into six dimensions, namely, structural, association, affectual, normative, consensual, and functional solidarity, which constituted the core independent variables of this study, as shown in Table 2. Due to the particularity of the one-child family structure, in the context of modernization, urbanization, and industrialization, adult children and their parents may show different characteristics in terms of emotions, attitudes, and actions. Structural solidarity refers to opportunities for daily intergenerational interaction, such as living arrangements and living distances, mainly based on the consideration of generational separation. Associative solidarity was assessed with the frequency of intergenerational family members’ contact, including face-to-face and non-face-to-face contact. Affectual solidarity refers to the positive relationship between family members, such as closeness, understanding, and trust, and the degree of reciprocity brought on by positive emotions. Normative solidarity refers to the degree to which family members identify with family roles and responsibilities, such as the evaluation of filial duty. Consensual solidarity refers to the degree of similarity in values, attitudes, and beliefs between parents and children. Functional solidarity was assessed by assessing whether parents had provided help to or received help from their adult child in financial support and instrumental help.

Except for normative solidarity and consensual solidarity, this study adopted the equal weighting method to calculate the scores of other dimensions. Because the measurement degree of the 11 indicators in the questionnaire was different, there were certain differences in the range of different dimensions. Except for functional solidarity, the higher the scores of other dimensions, the closer the family intergenerational relationship in that dimension. Functional solidarity mainly reflects the utility of child support and the role of intergenerational support relationships. The higher the score, the more intergenerational resources would flow to parents, and they played the role of “winners” in intergenerational interactions. The smaller it was, the more intergenerational resources would flow to child, and, accordingly, parents were “givers” in intergenerational interactions.

Is the family intergenerational relationship beneficial to the life satisfaction of only-child parents during the social transition? This study focused on the mechanism of family intergenerational relationships on life satisfaction and analyzed the influence of different relationship dimensions due to gender differences. The Satisfaction With Life Scale (SWLS) prepared by Diener et al. [49] was used to evaluate the living conditions of only-child parents in Chengdu. The five items were as follows: 1. In most ways my life is close to my ideal. 2. The conditions of my life are excellent. 3. I am satisfied with my life. 4. So far I have gotten the important things I want in life. 5. If I could live my life over, I would change almost nothing. In this study, a seven-point Likert scale was used to assign the degree of agreement. The seven-point scale was: 1 = strongly disagree; 2 = disagree; 3 = slightly disagree; 4 = neither agree nor disagree; 5 = slightly agree; 6 = agree; 7 = strongly agree. The scores of the above five questions were added and averaged to obtain the life satisfaction of the interviewees, with a value ranging from one to seven. Using the reliability and validity test, we found that the Cronbach coefficient of the scale was 0.855, the KMO statistic was 0.854, the chi-square value of the spherical Bartlett test was 839.675, and the associated probability was 0.000, indicating that the questionnaire data had good reliability and validity.

This study selected three types of control variables [22,24,39]. The first was the individual characteristics of only-child parents (including gender, age, years of education, marital status, hukou, health self-assessment, household income, and activity participation). The second was the individual characteristic variables of only-child (including gender, years of education, household income, and number of children). The third was that the life satisfaction of the only-child parents was related to external support, which was represented by the subjective evaluation of the community living environment.

The dependent variable was a continuous variable; multiple linear regression models and robust standard errors were adopted in the regression analysis to overcome the influence of heteroscedasticity. The collinearity test was performed for each variable, and the variance inflation factor was calculated. There was no multicollinearity problem. The moderating variable was “gender”, which was a dichotomous variable. In this study, group regression was used to compare the differences in the regression coefficients of multiple independent variables, and the Fisher’s Permutation test was used to verify the coefficient differences between groups, thereby determining the moderating effect of the gender. The above analysis process was based on Stata 15.1.

### 2.4. Thematic Analysis of Qualitative Research Data

In the qualitative analysis stage, this study mainly used the thematic analysis to analyze the interview materials, aiming to identify thematic patterns in the interview data. The basic steps drew on the experience of Braun and Clarke [50]. The thematic analysis was performed through a six-stage coding process to create meaningful patterns. These stages included becoming familiar with the data, generating initial codes, searching for themes, reviewing themes, defining and naming themes, and producing the final report. The thematic analysis could report on participants’ experiences, meanings, and realities, as well as examine the effects of events, realities, and experiences.

In order to ensure the trustworthiness of the qualitative analysis, this study adopted both member checks and a peer review in the qualitative research [51]. Structured questions were used to ask the respondents to confirm the analysis theory established by the research team. If the respondents agreed with the explanation given by the researcher, it provided confirmation evidence for the credibility of the conclusion. In the analysis process, we discussed the researcher’s explanation and conclusion with others, sought feedback, and solved the existing problems, which helped to provide some useful challenges and reflections.

## 3. Results

### 3.1. Life Satisfaction of Only-Child Parents

The life satisfaction of the only-child parents in Chengdu was 4.934. The t-test results showed a significant gender difference in life satisfaction, which was higher for men than for women (T = 2.877, *p* = 0.004). The life satisfaction of men was 5.094, whereas life satisfaction of women was 4.807. The group of middle-aged and elderly women was in a relatively disadvantaged state from the perspective of the whole life cycle, as a result of the accumulation of various social inequities due to gender, generation, and class. The subjects of this study were mainly born in the 1950s and 1970s, and their birth cohort population was less educated, especially when it came to the women. In the traditional family division of labor, women undertook more unpaid labor, such as family care [22]. Especially at this stage, their grandchildren’s care needs are relatively high, the pressure of family life is greater, and their physical and mental health may be worse. In terms of the economic situation, middle-aged and elderly women had lower economic income levels than men, enjoyed lower social security related to employment experience, faced greater economic risks, and had lower life satisfaction.

From the dual-gender perspective, there were differences in the life satisfaction of only-child parents depending on the child’s gender. Having a daughter could lead to greater life satisfaction, especially for women. First, considering the entire sample regardless of the gender of the respondents, compared with parents of sons (*M* = 4.812; *S* = 1.011), the life satisfaction of the sample group with daughters (*M* = 5.065, *S* = 0.994) was significantly higher, and it was significant at the 5% level. Second, from the dual-gender perspective, there was no significant difference in the impact of children’s gender structure on men’s life satisfaction (*p* = 0.465). However, the women’s life satisfaction from having a daughter (*M* = 4.994; *S* = 0.945) was significantly higher than from having a son (*M* = 4.637; *S* = 0.991), which was significant at the 1% level. Third, using the least significant difference method (LSD) to conduct multiple comparisons, the analysis found that when women gave birth to daughters, their life satisfaction was close to that of men, including two cases of giving birth to a son or daughter, and it was significant at the 1% level. Possible reasons were as follows: First, having a son is more financially demanding on the parents, including early education investments, as well as having to compete in the marriage market characterized by the gender imbalance through marriage houses and betrothal gifts [52]. Therefore, having a son substantially changed the lifestyle and consumption pattern in urban families. For women in a relatively disadvantaged economic position, the pressure and risk of family survival and development may be greater. Second, with the improvement of women’s economic status, women have gained greater power and autonomy in family decision making and actions. In addition to instrumental support, the financial and material support provided by daughters to their parents is also increasing [37]. In particular, the positive effect of intergenerational support of daughters may be more pronounced for women in an economically weak position. In addition, there were gender differences in emotional communication and expression. Considering that daughters were likely to be closer to their mothers, women who give birth to daughters were likely to have higher life satisfaction. Third, the gender division of labor in traditional families, such as family care, has distinctive feminized characteristics. In a traditional patrilineal family system, compared with a daughter, a mother and son are more likely to live together after the son marries, which requires more time and energy to take care of their spouses, sons, grandchildren, and even their parents. Because women assume multiple care roles [36], the related responsibilities increase significantly, and the pressure of the role may be more intense, whereas the harm to health was greater, such as increased prevalence, weakened immunity, and unhealthy behaviors, which may reduce life satisfaction.

### 3.2. The Intergenerational Relationship of Only-Child Families

Based on the subjective perspective of only-child parents, this study conducted a statistical analysis of the family intergenerational relationship with different genders of children (Table 3). Except for normative solidarity and consensual solidarity, sons and daughters had significant differences in other dimensions.

(1) Structural solidarity: Compared with the range, the relationship was weak, and sons provided higher satisfaction than daughters. In China, the occurrence of life events such as employment and marriage, the improvement of family economic conditions, and the awakening of individual consciousness greatly reduced the possibility of adult children living together with parents [27]. As a result, only-child families entered the empty-nest stage earlier than families with multiple children. The phenomenon of intergenerational cohabitation was not prominent in Chengdu, which accounted for 32.84%, but the intergenerational space distance was relatively small, and the proportion of co-living in Sichuan province was 68.63%. In recent years, the popularization of private cars and the construction of transportation infrastructure, such as the Chengdu metro, the Chengdu–Mianyang–Leshan high-speed railway, and the Chengdu–Chongqing high-speed railway, have changed the travel mode and efficiency, effectively reducing the spatial and temporal distance between generations. As one interviewee stated: “My son works in Chongqing, the multiple unit trains and high-speed trains are very convenient, and there are many trips every day” (Interviewee: male, 56 years old, middle school teacher). Gender differences in children mainly come from the patriarchy [18,31]. Sons are more likely to live with their parents after marriage, and their choice of employment location may also take into account the spatial distance from their parents. The daughter is more likely to live with their husband; thus, the daughter is not as good as the son in the structural relationship.

(2) Association solidarity: Compared with the range, the relationship was stronger, and sons provided higher satisfaction than daughters. An important reason was that sons were more likely to choose intergenerational cohabitation after marriage and have more daily contact with their parents [53]. Consequently, the association solidarity was greater. In the case of intergenerational separation, due to the influence of Chinese traditional culture, paternal grandparents may give more life care or emotional care to grandchildren than maternal grandchildren and parents may also have more frequent contact with their son indirectly. With the outbreak of the COVID-19 pandemic, which is a public health emergency, many countries, including China, adopted different levels of restrictions and control measures according to the epidemic situation to mitigate adverse events. Accordingly, face-to-face intergenerational communication may have been reduced. Fortunately, mobile phones and chat tools such as WeChat and QQ have played a great compensatory role and provided support for interactions and connections among intergenerational family members, reducing time and economic and opportunity costs. However, our findings from the semistructured interviews revealed that sons were less proactive than daughters in daily contact. For example, “If he has free time, he will also call us, but it is relatively rare. The main thing is that we take the initiative to contact him and ask about the granddaughter situation” (Interviewee: male, 63 years old, retired worker).

(3) Affectual solidarity: Compared with the range, the relationship was stronger, and sons provided lower satisfaction than daughters. Influenced by the traditional division of roles within the family, women were solidified as family caregivers and more likely to be the maintainers of family emotions. Only-child parents subjectively believed that daughters provided more emotional support in daily life and daughters were more likely to be considerate to their parents, thus, maintaining strong emotional unity. For example, “My son will call us on weekends and holidays to ask about our family and care about our health” (Interviewee: female, 53 years old, primary school teacher). In addition, “She also asks us for advice on important family matters. Of course, we also seek her opinion on many things” (Interviewee: male, 56 years old, individual business). This difference in the gender structure of children is obviously different from the traditional patrilineal society that pays more attention to the patriarchal line, focusing on the father–son relationship, where gender roles trump actual emotions [14]. This shows that under the uniqueness of the number of children, the relationship of the daughter and original family is characterized by stability and intimacy in the emotional dimension, which is no longer temporary and weak.

(4) Functional solidarity: The functional utility of adult children appeared to be de-gendering, even demonstrated a tendency of daughter endowment, which is different from the cultural logic of “raising children to prevent old age” in China [37]. A realistic reason for this positive change in roles is that the only-child policy further promoted the transformation of the family inheritance system. Adult children have the same obligations to their parents, and daughters actively built functional relationships as an inherent stipulation based on legal norms and ethical responsibilities, which evolved into a rigid responsibility. This was also related to China’s reform and opening up and the process of marketization. In China, women returned to society and equally participate in sociopolitical and economic life. The improvement of their socioeconomic status has prompted women to have sufficient dominance in their families [43]. In this case, it also promotes the manifestation of daughters’ responsibilities, values, and contributions to their families of origin. Through the semistructured interviews, we found that functional relationships were more about active selection and mutual assistance based on needs [8], rather than on regulations under rigid responsibilities. In general, only-child parents were less dependent on their children in functional relationships, which was mainly related to the life cycle stage of the two generations. Urban only-child parents were mainly middle-aged or young elderly, with a better health and economic status, and strong autonomy and independence, while the only-child was affected by multiple influences such as urban competition pressure and modern family division of labor. Accordingly, inverse dependencies may form in functional relationships. Considering financial support, parents’ economic dependence on their adult children was considerably weakened: “My son is repaying the mortgage every month, the grandson’s education costs are also high, and the family is under great financial pressure. Sometimes he gives us some money, we don’t really use his money and just help him manage it and give it to him when he needs it” (Interviewee: male, 55 years old, individual business). “My daughter is married and has her own family. Sometimes she gives money to us, but we will not accept it, we still have fixed income to cover our daily expenses” (Interviewee: female, 56 years old, housewife). “As long as we don’t suffer from serious illnesses and don’t have huge expenses, we don’t need him to give money” (Interviewee: male, 55 years old, individual business). Considering labor support, many parents actively or passively supported their child: “Parents help to take care of grandchildren as a matter of course, it’s a custom passed down from the ancestors, which can ease their burden” (Interviewee: female, 56 years old, housewife). “When your children need care support and you don’t give help, other people will inevitably gossip” (Interviewee: male, 63 years old, retired worker).

Compared with the range, the normative solidarity was stronger, whereas the consensual solidarity was weaker, and there were no significant gender differences. Only-child grew up in a period of rapid social transformation, and their socialization development was different from those of multiple children. With the rise of individualized values, only-child may pay more attention to self-expression and self-worth. There were changes in value systems, behaviors, and lifestyles, and there were large differences between generations. However, the concept of filial piety among only-child had not faded.

### 3.3. Associational Mechanism of Family Intergenerational Relationship on Life Satisfaction

In China, family intergenerational relationships had a critical impact on life satisfaction of only-child parents (Table 4). The control variables basically conformed to existing research conclusions and were not repeated here.

(1) Structural solidarity: Structural solidarity had no significant effect on life satisfaction. With the process of modernization, people’s behavior, lifestyle, and value system have undergone substantial changes and weakened the existing foundation of the traditional Chinese family [27]. In urban China, the improvement of urban housing conditions and material living standards were followed by the awakening of individualism, such as the pursuit of privacy, independence, and freedom. Family structures tend to be miniaturized in residential forms, and intergenerational separation has become an autonomous choice in modern urban society. Within the family, intergenerational members negotiate and trade-off multiple goals, such as economic costs, care needs, and personal or family development [21]. Moreover, they make conscious choices about structural relationships, such as lifestyle or living arrangements in certain contexts. In the family form, choosing the type of structural relationship becomes a strategic choice to maximize the economic utility of the family. Different from the classical family modernization theory, the urban family structure in Chengdu did not move toward nucleation and nonkinship. In the survey sample, the only-child families showed a preference for being separated, while generational members were not far away, which allowed for greater autonomy and flexibility. This enabled the members to maintain privacy, but also helped build a family support network, which provided timely mutual assistance and effective emotional communication and had strong risk tolerance and coping capabilities. The regression coefficient was positive but not significant for women. One possible explanation is that under patriarchal cultures, parents have greater structural solidarity with children, such as living with or nearby their married children. Mothers may be given the role of caregiver, who invest in housework, emotional expenditure, and childcare, using considerable family resources and leisure time, especially considering that most mothers are still in the labor market and work–family conflicts are relatively common. In stem families, there may also be tensions in the relationship between the mother-in-law and daughter-in-law, which would undoubtedly exacerbate the reduction in women’s life satisfaction. From this perspective, it is not difficult to understand that women who bear daughters have higher life satisfaction. In general, in terms of structural solidarity, family separation in the spatial form did not have a significant impact on life satisfaction.

(2) Association solidarity. According to the social escort theory [54], the individual social relationship is a self-centered differential order pattern. In the process of aging, individuals faced a decline in physiological functions and the loss of social roles, whereas the social networks continued to shrink based on kinship. Especially in China, the social network is based on blood relationships. Compared with nonfamily members, individuals become increasingly dependent on family members, such as children. Theoretically, the pressure of family life is greater in urban society, especially for the floating population which lacks local household registrations (hukou), separated from the original geographical relationship. Relatively frequent daily contact with children can alleviate loneliness, anxiety, and depression, which in turn considerably improves life satisfaction. In models 1–3, the regression coefficients were positive, but not significant. As a result of the COVID-19 pandemic [55], individuals were isolated from certain social relationships and forced to change the way they interacted with others. Within the family, face-to-face communication between parents and children was greatly reduced or even absent. Because modern communication technologies such as the telephone and internet have broken through the limitations of space, intergenerational interactions have remained relatively frequent. However, the extent to which this compensatory or substitution effect plays a role is worth considering. This study mainly examined the impact of contact frequency, and its communication quality also needs further analysis. In addition, the parents in the survey sample were young and had frequent connections outside the family, and the positive effects of parent–child connections may have been relatively limited. Because of social roles, social networks, and age structures, intergenerational connections had no significant impact on the life satisfaction of urban only-child parents.

(3) Affectual solidarity. Family intimacy was the core factor of life satisfaction, especially for women. The Western modernization theory assumes that with the growth of individualism and correlation, the decline of patriarchy has taken part. More precisely, the father–son emotional connection may continue to weaken, after which the spouse relationship is expected to become the most important family relationship in contemporary China [5]. The regression analysis results showed that intergenerational members actively built family intimacy through language and behavior, and satisfied each other’s emotional demands, which helped improve life satisfaction. The reason is that the stronger the emotional relationship, the easier it is for individuals to feel valued, needed, and accepted in the family, and the individuals’ self-efficacy may be stronger. Furthermore, the positive emotional relationship was conducive to realizing a common understanding and trust and reducing intergenerational conflicts, thus, easing family conflicts and making intergenerational relations more harmonious. Among them, affectual solidarity was more positive for women. Corresponding to the previous analysis, daughters had stronger affectual solidarity than sons, which is a possible explanation for the higher life satisfaction of parents who raised daughters, especially for women. Affected by the gender division of labor in traditional families, in which the outside is managed by men and the interior by women, mothers have more frequent emotional communication with children. In daily sexual interactions, their emotional relationships have a direct effect on women’ psychological and physical health, as well as life satisfaction. In addition, it is also related to biological characteristics, such as menopause [56]. Most of the women were between the ages of 50 and 60 in the survey sample. Therefore, the fluctuation or decrease in sex hormones caused a series of autonomic nervous system dysfunctions, accompanied by upset, irritability, fear, anxiety, and other psychological symptoms. For women, adequate intergenerational emotional communication and positive intergenerational emotional experience were conducive to enhancing the adaptive capacity and adjustment ability, slowing down negative emotions and, thus, improving life satisfaction.

(4) Normative solidarity. The degree of filial piety had a significant positive effect on the life satisfaction of only-child parents, but there was a gender difference. At present, China’s institutional arrangements and policy system for dealing with aging are not mature and perfect. The family-based pension model, in which adult children provide support to their parents, has become an institutionalized strategy to avoid old-age risks [15]. In the Chinese context, the more filial piety of the adult children, the more intergenerational support they may provide, including materials, services, and emotions, which increase the parents’ pension capital and old-age well-being [57]. Hence, children’s filial piety had a critical impact on parents’ life satisfaction from the perspective of pension risk aversion; however, there were gender differences related to this effect. A possible explanation is that under individualized and structural stress conditions, influenced by ethical responsibilities, only-child parents have relatively low filial piety expectations and have a relatively high degree of filial piety of their children. However, influenced by traditional patrilineal culture, men attach great importance to patriarchal power and filial piety of children, especially the obedience and no violation of adult children may have had some positive effects on their life satisfaction. In contrast, with the construction of new filial piety culture, the explanatory power of children’s filial piety on women’s life satisfaction is constantly weakening in urban China.

(5) Consensual solidarity. The impact of consensual solidarity on life satisfaction differed by gender and was beneficial for men. The character, personality, temperament, and preference of the parents were transmitted indirectly or directly to their offspring, with obvious inheritance within the family [58]. Different from Western countries, the family of origin plays a more obvious role in individual socialization, and the inheritance relationships, such as value judgment and conduct code and moral code, almost run through the whole life course of children. Under this logic, the stronger the intergenerational transmission effect, the more similar the cognitive styles and behavioral patterns between parents and children and the greater the homogeneity; thus, family intergenerational relationships may be more stable. The regression results showed that the consensual solidarity had a significant impact on the life satisfaction of men, but not on women, and there was a gender moderating effect. This was explained as follows: First, under the influence of social transformation, the proportion of intergenerational cohabitation decreased. The weakening of consensual solidarity, such as intergenerational differences in living habits, family values, and cultural orientations, may alleviate or avoid conflicts and contradictions in daily life due to spatial separation and allow maintaining close family relationships. Second, in urban society, the high degree of social mobility led to a decline in patriarchal culture, while the freedom of family members increased, and the binding force on the construction of individual consciousness weakened. For parents, their urbanization led to significant changes in values, cultural norms, and ways of thinking. In daily life, the constraints of parents on the consensual relationship between their children were constantly reduced. For example, the objective existence of the “generation gap” has become a relatively common social phenomenon in China. The reason for the gender-moderating effect is that the gender power structure of patriarchal culture has not undergone substantial changes [59]. Traditional oriental society attaches great importance to the family inheritance system centered on patrilineal blood. In the ideological field, men pay more attention to the value and cultural similarity under the blood link to achieve psychological integration and family continuity. For men, the stronger consensual solidarity, the more significant of intergenerational transmission effect and the greater the sense of satisfaction, fulfillment, and belonging.

(6) Functional solidarity. The effect of functional solidarity on life satisfaction was not significant, and it was mainly related to the life cycle stage of the only-child family. In urban society, the economic independence of only-child parents was relatively good, and the degree of economic dependence on their children was lower compared to traditional societies. As revealed by the semistructured interviews, although the net economic flow is from children to parents, it consists of symbolic expenditures such as holidays and birthdays and it does not have a rigid logic. In urban China, the competitive pressures and living costs faced by Chinese urban families are much higher. In many families, downward intergenerational economic support continues to emerge, which is reflected in events such as marriage, house purchase, and entrepreneurship to minimize the survival pressure of adult children [60]. This large amount of intergenerational economic support may affect the quality of life of parents to a certain extent and even lead to intergenerational conflicts. However, we found that this was mainly due to parents adopting active strategies and taking the family as a unit to maximize family interests, which reflected the corporate model in intergenerational supportive behaviors revealed by the semistructured interviews. Furthermore, only-child parents at this stage were relatively young. In instrumental support, it is more common that only-child become increasingly dependent on their parents’ labor services, most related to the childcare needs, especially for sons to achieve family continuity [61]. In the life cycle, not all of the only-child parents were in the absolute support stage and were influenced by traditional family culture; they had autonomy and initiative in functional interaction. Therefore, in urban society, parents play the role of “winners” in intergenerational interactions, and their role in improving their life satisfaction is not significant. Of course, parents being in the role of the “giver” does not necessarily mean low life satisfaction. Consistent with this conclusion, we found a trend different from traditional family cultural norms using the semistructured interviews. When medical expenses, such as chronic diseases and serious diseases, were excluded, many urban only-child parents were influenced by modern urban culture and had a negative attitude toward relying on their adult children’s financial support to live, which has begun to be stigmatized. More specifically, excessive material dependence on children tends to become a denial of self-survival value. This behavioral logic is no longer equivalent to the intergenerational feedback model in traditional Chinese culture, as illustrated by the following quotations “It is shameful to ask children for basic living expenses” (Interviewee: male, 52 years old, Didi driver) and “I’m so useless? It’s not good to say it out” (Interviewee: female, 55 years old, individual business).

## 4. Discussion

Under the increasing urbanization, population aging, and modernization, this study took only-child families in Chengdu as the research object, evaluated the intergenerational relationships of Chinese urban families from the perspective of intergenerational solidarity, and investigated the impact of family intergenerational relationships on life satisfaction of only-child parents. It provided a new perspective for effectively responding to the post-aging society. This perspective broke through the previous paradigm of life satisfaction research by exploring the life satisfaction of middle-aged and elderly parents in the process of changes in family structure and intergenerational relations. Moreover, this perspective can play a critical practical role in creating a positive family intergenerational relationship, consolidating the traditional family-based pension model in China, and achieving active and healthy aging. In contrast to traditional Chinese culture, this study found that some urban only-child parents began to stigmatize their dependence on their children’s financial support for life, which may reverse to a denial of self-worth. The mechanism behind this needs to be further analyzed in future studies. For a long period, the family was the foundation of all the institutional constructions of East Asian societies. The only-child parents entering the ranks of older adults or oldest-old are likely to become an increasingly large social group in the coming decades. In the microscopic field of families, Chinese society needs to pay attention to the particularity of only-child families, construct positive family intergenerational relations, consolidate the foundation of family pension, properly solve the problems of the survival and development of the elderly, actively explore public policies to support the family, and seek for the reconstruction of family policies.

This study still had several limitations. First, considering the measurement of family intergenerational relationships, there was a lack of direct observation indicators for contradictions or conflicts, failing to fully reflect the complexity of contemporary urban family intergenerational relationships. Second, this study was based on the perspective of only-child parents, which neglected the possible differences of intergenerational identity in the relationship dimension. The ideal situation would be to match two generations from the same family. Due to the difficulty and cost of the investigation, there was a lack of investigation of the corresponding offspring samples, and the analysis conclusion may be biased. Supplementary studies can be conducted based on typical sample families in the future. It is highly recommended to match parents and children within the same family. Due to the research difficulty, cost, and related lack of corresponding offspring samples, there may be some deviations in the analysis conclusions. In the future, supplementary research can be carried out based on typical sample families. Third, this study took Chengdu as a case, mainly examining urban only-child families. There was a lack of a comparative analysis of rural area samples. The extent to which the research conclusions can be inferred to other areas in China needs further testing.

## 5. Conclusions

Our results showed gender differences in life satisfaction of urban only-child parents in Chengdu. More specifically, women’s life satisfaction is lower than men. The analysis from the dual-gender perspective found that when women gave birth to daughter, their life satisfaction was close to men, including two cases of men having a son or a daughter. This is undoubtedly different from the traditional Chinese culture of “raising a son to prevent old age” and the preference for boys.

Urban only-child families maintained close family relationships in Chengdu, and they have not loosened, fractured, or disintegrated, as predicted by Western modernization theories. However, there were significant dimension and gender differences in family intergenerational relations. Structural solidarity is relatively weak, whereas association solidarity and affectual solidarity were stronger. In functional solidarity, the functional utility of children showed a trend of de-gendering, even daughter endowment, which considering that the utility and value of daughters to the original family have emerged. In normative solidarity and consensual solidarity, there were no gender differences of children in only-child families.

In different dimensions, the family intergenerational relationships had a different influence on the life satisfaction of only-child parents. In urban society, affectual solidarity was an important dimension of life satisfaction, and it had a greater impact on women’s life satisfaction. In terms of normative solidarity and consensual solidarity, “gender” had a moderating effect, which was beneficial to improving men’s life satisfaction, but the same did not hold true for women. Considering a reduction in structural solidarity, formal spatial separation did not have a significant effect on life satisfaction. The effect of association solidarity on life satisfaction was not significant, possibly due to the reduction or absence in face-to-face intergenerational communication during the COVID-19 pandemic. Only-child parents were still relatively young, and their utility may have been disturbed by the external connectedness of the family. Functional solidarity had no significant influence on life satisfaction, which was mainly related to the traditional family culture and the life cycle stage of the only-child families.

## Figures and Tables

**Table 1:** Demographic characteristics.

Demographic Characteristics	Variable Assignment	Mean (Standard Deviation)
Gender	Male = 1; female = 0	0.444 (0.497)
Age	Aged 60 and above = 1; Under the age of 60 = 0	0.289 (0.454)
Years of education	Primary school and below = 6; middle school = 9; high school/vocational school = 12; two-/three-year college/associate degree = 15; four-year college/bachelor’s degree and above = 16	9.176 (2.626)
Marital status	Married = 1; not married = 0	0.909 (0.288)
Hukou	Non-agricultural hukou = 1; agricultural hukou = 0	0.547 (0.498)
Health self-assessment	Very good = 5; good = 4; fair = 3; poor = 2; very poor = 1	3.397 (1.032)
Household income	Less than 100,000 CNY = 1; 100,000~200,000 CNY = 2; 200,000 CNY and above = 3	1.637 (0.635)
Activity participation	Continuous variable	3.485 (2.056)

**Table 2:** Measurement indicators of intergenerational relations under the intergenerational solidarity theory.

Dimension	Item	Option
Structural solidarity	What is the living distance between you and your children?	7 = live together; 6 = in the same community; 5 = on the same street; 4 = in the same district; 3 = in the same city; 2 = in the same province; 1 = in a different province
	How long is your trip to where your children live?	7 = live together; 6 = within 30 min; 5 = within 1 h; 4 = within 2 h; 3 = within 3 h; 2 = within 4 h; 1 = 4 h and above
Association solidarity	How often do you and your children see each other?	7 = almost every day; 6 = 3–4 times a week; 5 = 1–2 times a week; 4 = 1–2 times a month; 3 = several times a year; 2 = once a year; 1 = almost never
	How often do you and your child communicate with each other by phone, online, and video?	7 = almost every day; 6 = 3–4 times a week; 5 = 1–2 times a week; 4 = 1–2 times a month; 3 = several times a year; 2 = once a year; 1 = almost never
Affectual solidarity	Do you feel emotionally close to your children?	5 = very close; 4 = somewhat close; 3 = uncertain; 2 = not very close; 1 = very unapproachable
	Do you think you get along well with your children?	5 = very good; 4 = somewhat good; 3 = uncertain; 2 = not very good; 1 = very bad
	Do you think your children are willing to listen when you want to share your thoughts or difficulties with your children?	5 = very willing; 4 = somewhat willing; 3 = uncertain; 2 = not very willing; 1 = very reluctant
Normative solidarity	Do you think your children are filial?	5 = very filial; 4 = somewhat filial; 3 = uncertain; 2 = somewhat unfilial; 1 = very unfilial
Consensual solidarity	Do you think you have similarities with your children in terms of values, attitudes, and beliefs?	5 = strongly agree; 4 = somewhat agree; 3 = uncertain; 2 = disagree; 1 = strongly disagree
Functional solidarity	What is the net flow of the intergenerational economy?	3 = child flow to parents; 2 = no obvious flow; 1 = parents flow to child
	What is the net flow of intergenerational labor services?	3 = child flow to parents; 2 = no obvious flow; 1 = parents flow to child

**Table 3:** Gender test of family intergenerational relationships.

Child Categories	Structural Solidarity	Association Solidarity	Affectual Solidarity	Normative Solidarity	Consensual Solidarity	Functional Solidarity
Sons	4.384 ± 2.565	5.206 ± 1.268	3.946 ± 0.669	4.095 ± 0.775	3.493 ± 0.958	1.962 ± 0.542
Daughters	3.779 ± 2.025	4.939 ± 1.198	4.176 ± 0.564	4.188 ± 0.707	3.477 ± 0.739	2.216 ± 0.485
T test	2.652 **	2.187 *	−3.735 ***	−1.264	0.186	−4.969 ***
Range	1~7	1~7	1~5	1~5	1~5	1~3

All data are expressed as means ± SD in the first and second row of this table; * *p* < 0.05; ** *p* < 0.01; *** *p* < 0.001.

**Table 4:** Results of regression model.

Variables	Model 1 All Sample		Model 2 Male		Model 3 Female		Moderating Effect Test of Gender
	Coefficient	Robust Standard Error	Coefficient	Robust Standard Error	Coefficient	Robust Standard Error	
Core independent variable							
Structural solidarity	−0.008	0.034	−0.026	0.061	0.020	0.043	−0.046
Association solidarity	0.050	0.066	0.041	0.123	0.034	0.074	0.007
Affectual solidarity	0.403 ***	0.097	0.266 **	0.125	0.529 ***	0.129	−0.263 *
Normative solidarity	0.153 **	0.072	0.271 **	0.128	0.056	0.087	0.215 *
Consensual solidarity	0.064	0.045	0.181 **	0.072	0.032	0.067	0.149 **
Functional solidarity	0.042	0.071	−0.147	0.105	0.056	0.111	−0.203 *
Control variable							
Personal characteristics							
Gender (reference group: female)	0.230 ***	0.068					
Age (reference group: under the age of 60)	0.057	0.080	−0.103	0.129	0.152	0.100	
Years of education	0.011	0.020	0.021	0.029	−0.008	0.028	
Marital status (reference group: not married)	0.197	0.157	−0.014	0.256	0.250	0.177	
Hukou (reference group: agricultural hukou)	0.189 **	0.089	−0.070	0.127	0.398 ***	0.127	
Health self-assessment	0.127 ***	0.041	0.107 *	0.062	0.132 ***	0.047	
Household income (reference group: less than 100,000 CNY)							
100,000~200,000 CNY	0.154 *	0.088	0.146	0.158	0.063	0.099	
200,000 CNY and above	0.176	0.135	0.164	0.209	0.025	0.197	
Activity participation	0.039 **	0.019	0.021	0.029	0.069 ***	0.026	
Child characteristics							
Child’s gender (reference group: female)	−0.137 *	0.070	−0.060	0.113	−0.208 **	0.096	
Child’s years of education	0.036 **	0.018	−0.002	0.030	0.068 ***	0.020	
Child’s household income (reference group: 100,000~200,000 CNY)							
Less than 100,000 CNY	−0.252 **	0.108	−0.628 ***	0.171	−0.097	0.139	
200,000~300,000 CNY)	0.142 *	0.074	0.131	0.133	0.203 **	0.098	
300,000 CNY and above	0.091	0.150	−0.024	0.210	0.251	0.258	
Number of children	−0.131	0.083	−0.261 **	0.114	0.003	0.113	
Community characteristics							
Community environmental assessment	0.151 ***	0.037	0.163 **	0.067	0.131 ***	0.043	
Constant	0.145	0.382	1.672 **	0.786	−0.526	0.440	
F-value	50.03		16.28		42.33		
P-value	0.000		0.000		0.000		
R-squared	0.635		0.640		0.694		

* *p* < 0.1; ** *p* < 0.05; *** *p* < 0.01.

## Data Availability

The data that support the findings of this study are available from the corresponding author upon reasonable request.
